# Weak isolation by distance in *Diaperis boleti* , a fungivorous saproxylic beetle

**DOI:** 10.1093/jis/14.1.109

**Published:** 2014-08-12

**Authors:** Andrzej Oleksa

**Affiliations:** Institute of Experimental Biology, Kazimierz Wielki University, Chodkiewicza 30, 85-064 Bydgoszcz, Poland

**Keywords:** spatial genetic structure, spatial autocorrelation, AFLP, inbreeding, saproxylic beetles

## Abstract

Living in unstable habitats is expected to decrease the intensity of isolation by distance in populations through the need for frequent movements of individuals. Insects associated with fruiting bodies of fungi therefore are supposed to have weak spatial genetic structure of populations compared with those living in more stable habitats. With the use of an amplified fragment length polymorphism technique, this study investigated the isolation by distance, inbreeding, and genetic diversity in *Diaperis boleti* (L.) (Coleoptera: Tenebrionidae), a fungivorous saproxylic beetle that inhabits sporocarps of *Laetiporus sulphureus* (Bulliard) Murrill (Polyporales) on trees growing in highly-fragmented agricultural landscapes. Isolation by distance was tested with spatial autocorrelation analysis of kinship (individual-based approach) and correlating matrices of genetic and geographic distances with the Mantel test (population-based approach). These results were compared with the results obtained for saproxylic beetles living in the same landscape but differing in ecological preferences. It was shown that the species dependent on sporocarps of wood-decomposing fungi had higher variability, lower individual inbreeding, and less intensive isolation by distance pattern than saproxylic beetles living in tree hollows. It was also demonstrated that spatial autocorrelation analysis of kinship is a more sensitive approach for detecting fine-scale spatial genetic structure than the Mantel test.

## Introduction

The abilities of organisms to disperse among subpopulations and to colonize distant habitat patches are important for metapopulation survival, especially in landscapes subjected to a strong fragmentation ( Clobert et al. 2004 ). Therefore, the evolution of dispersal traditionally has attracted the attention of conservation biologists ( Macdonald and Johnson 2001 ). Theoretical models (e.g., Travis and Dytham 1999 ) have shown that dispersal strategy of an organism could be related to environmental predictability and habitat longevity. Therefore, species confined to more ephemeral habitats are predicted to be less susceptible to habitat fragmentation than their relatives that occupy habitats with low temporal but high spatial variability. Thus, living in stable habitats should drive evolution of dispersal toward lower rates, whereas living in habitats with fast turnover rate is expected to stimulate evolution of high dispersal capacities ( Roff 1975 ).

Unfortunately, empirical results showing a link between ecological specialization and dispersal abilities of insects are scarce (for examples, see Nilsson and Baranowski 1997 , Peterson and Denno 1998 , Oleksa et al. 2012a ). The shortage of data could be attributed to methodological constraints related to dispersal studies in insects. Traditional direct methods, such as mark‒release‒recapture or radio-tracking are applicable for larger species (e.g., Hedin et al. 2007 , Dubois et al. 2010 , Drag et al. 2011 , Chiari et al. 2012 ); for most insects, only indirect, molecular methods are available (for review, see Ranius 2006 ). Thus, analyses of spatial distribution of genetic diversity in the context of effective gene flow within and among populations should allow for the inference about dispersal in small-bodied insects.

Insects connected with diverse dead-wood habitats (i.e., saproxylic insects, Speight 1989 ) are a good model group in which to study the effect of specialization on dispersal evolution ( Ranius 2006 ) because of the many different forms of microenvironments related with dead or dying trees, for example, tree hollows with wood mould, snags, branches, or fruiting bodies of wood-decomposing fungi ( Grove 2002 , Stokland et al. 2012 ). All these resources are characterized by varying durability. For example, wood mould in large tree hollows can last for decades and support tens or even hundreds of generations of insects, whereas the fruiting bodies of wood-decomposing fungi could be placed on the opposite end of the durability spectrum ( Stokland et al. 2012 ). Some of fruiting bodies last for only days or weeks, and dependent insects must colonize them and complete their life cycle within a restricted time period. Because of high turnover rate of habitat patches, high dispersal and colonization ability are expected to enable the survival of the metapopulation of fungivorous insects.

In this study, AFLP (amplified fragment length polymorphism) genetic markers were used to examine isolation by distance (IBD) of *Diaperis boleti* (L.) (Coleoptera: Tenebrionidae), a beetle that inhabits fruiting bodies of wood-decomposing fungi. IBD is a phenomenon characterized by increasing genetic divergence due to decreasing gene flow with increasing geographic distance between individuals or populations ( Wright 1943 ). In a previous study, Oleksa et al. (2012a) found that beetles living in tree hollows are characterized by a strong IBD pattern. According to the theoretical predictions, organisms that depend on more ephemeral resources should exhibit a weaker IBD. Moreover, overall high dispersal rate of the species is expected because the animals are relatively small and macropterous and potentially can disperse by passive and active flight. It is likely that spatial genetic structure is weaker for saproxylic beetles living on fruiting bodies of fungi as compared with those living in more stable dead-wood habitats. Also, the presumably high dispersal ability of *D. boleti* should result in decreased level of inbreeding compared with the presumably less mobile species. To investigate these questions, IBD and inbreeding in *D. boleti* were studied.

## Materials and Methods

### Study Species

*D. boleti* is a widespread species, occurring in almost all of Europe (except extreme northern areas); it is reported also from Northern Africa and the Middle East ( Burakowski et al. 1987 ). The species lives in sporocarps of fungi growing on mainly deciduous trees (rarely on conifers). *D. boleti* mostly develop in live mature fruiting bodies, but they also are able to use the dry remnants of older fruiting bodies ( Schigel 2011 ). As the host fungi, the following species of the order Polyporales were reported ( Burakowski et al. 1987 ): *Laetiporus sulphureus* (Bulliard) Murrill (Fomitopsidaceae), *Piptoporus betulinus* (Bulliard) P. Karsten (Fomitopsidaceae), *Fomitopsis pini-cola* (Swartz) P. Karsten (Fomitopsidaceae), *Fomes fomentarius* (L.) Fries (Polyporaceae) (cf. http://www.mycobank.org ). The complete life cycle takes one year; the beetles emerge in the summer and survive the winter as adults ( Burakowski et al. 1987 ). Because the durability of sporocarps of the main host ( *L. sulphureus* ) is also one year, beetles must colonize a new habitat patch every year.

### Study area

**Figure 1.**
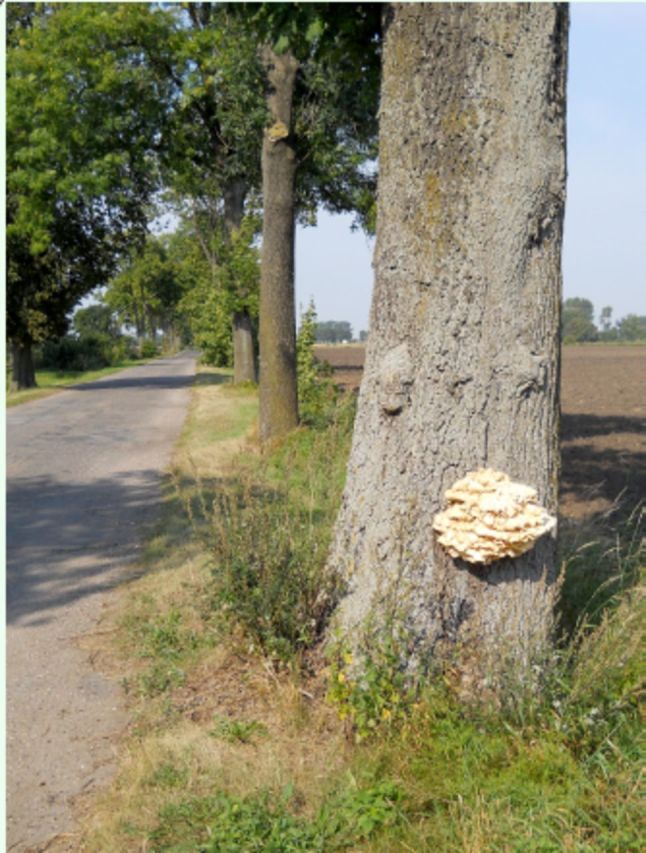
Sulphur polypore *Laetiporus sulphureus* growing on roadside tree in a rural avenue. High quality figures are [available online.

**Figure 2.**
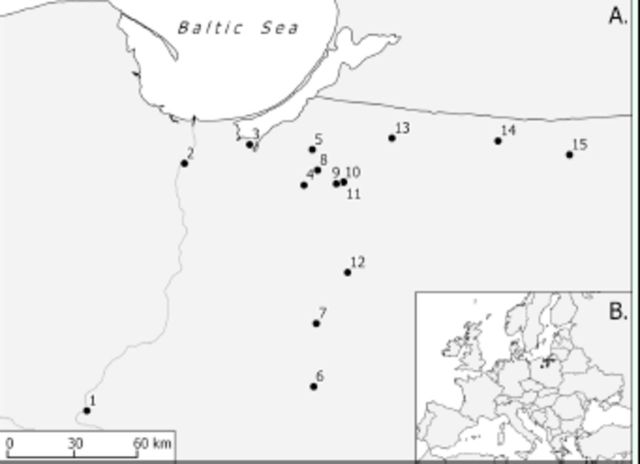
Collection sites of *Diaperis boleti* in northern Poland (A) and location of the area under study in Europe (B). High quality figures are available online.

For this study, samples of *D. boleti* were collected from fruiting bodies of *L. sulphureus* growing on roadside trees in rural avenues ( Fig. 1 ) in northern Poland in the area between the Lower Vistula Valley and the Great Masurian Lakes, between 18°56′ and 21°43′ E and 53°16′ and 54°19′ N ( Fig. 2 ). The study area is dominated by an agricultural landscape rich in historical avenues with trees planted along roads (average density of avenues was estimated as 0.31 ± 0.04 km km ^-2^ ; Oleksa et al. 2012b ). The most common tree species in the se avenues is *Tilia cordata* (≈50% of all trees in alleys), followed by *Quercus robur* , *Fraxinus excelsior* , and *Acer platanoides* (each species represents ≈10% of all trees), and *Betula pendula, Carpinus betulus* and *Salix alba* (each species represents 2‒3% of all trees). Other trees ( *Alnus glutinosa* , *Pyrus communis* , *Aesculus hippocastanum* , *Acer pseudoplata-nus* , *Ulmus glabra* , *Populus* sp., *Malus* sp., *Acer saccharinum* , *Pinus sylvestris* , *Picea abies* , *Salix caprea* ; listed in order of decreasing abundance) have a share of <1% (for the detailed list, see Oleksa et al. 2007 ). All samples of *D. boleti* were gathered in summer months of 2009 and 2010.

### Sampling design

Sampling was designed to quantify the spatial structure of genetic data over different spatial scales, from a single habitat patch (single sporocarp) to >200 km. Samples were collected to provide a large number of pairwise comparisons between genotypes for different geographical distances. Although the goal was to collect at least 10 individuals from each sporocarp, in most cases a smaller number of individuals were encountered and so as many as possible were collected ( Table 1 ). To avoid collecting highly related groups of individuals consisting of offspring of limited number of parents, insects were collected from fresh (newly colonized) sporocarps. Altogether, samples from 136 individuals of *D. boleti* from 15 sporocarps were collected in vials with 90% ethanol and preserved at ‒20°C until DNA extraction.

**Table 1.**
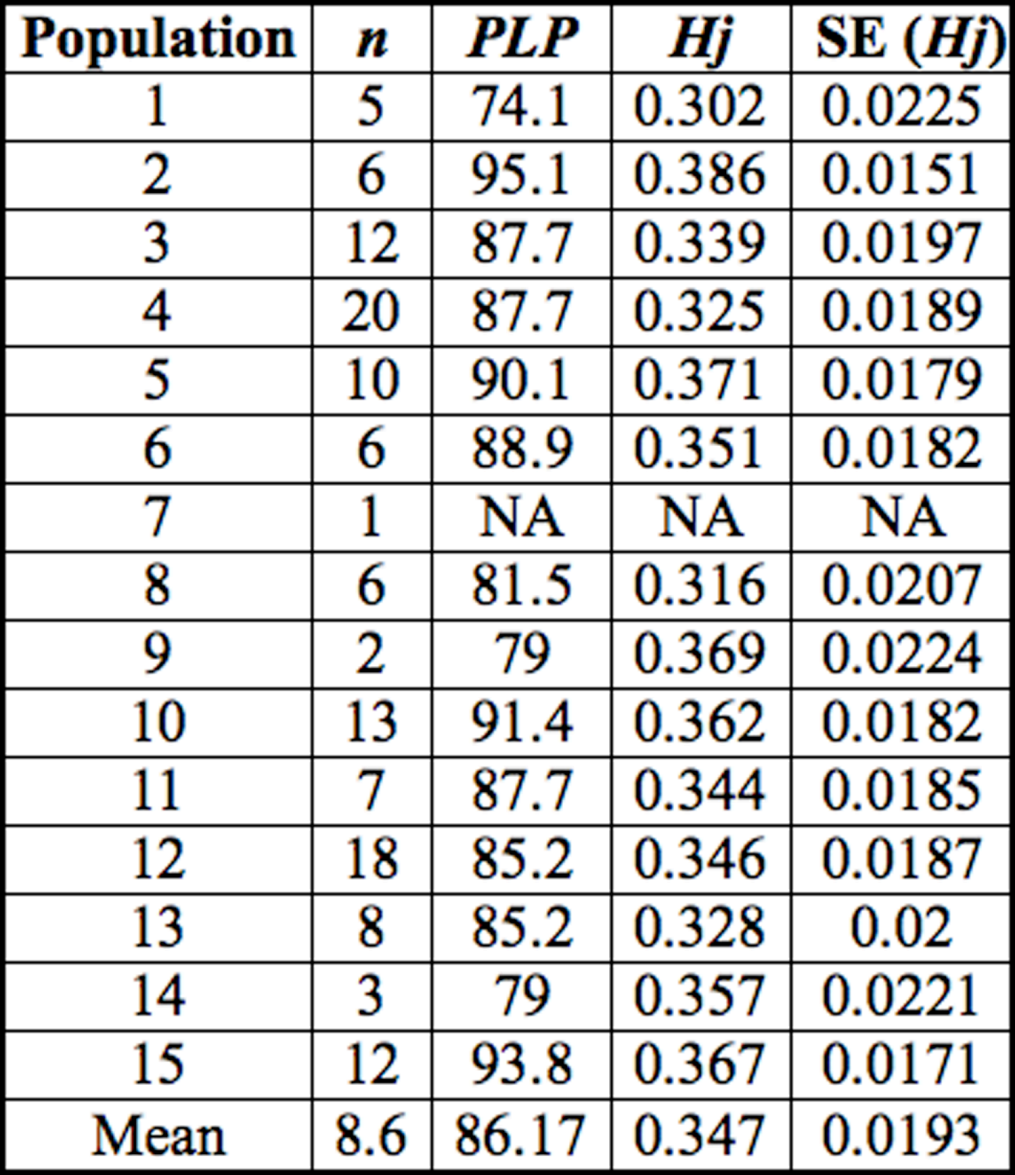
Proportion of polymorphic loci ( *PLP* ) at the 5 % level and expected heterozygosities under Hardy–Weinberg genotypic proportions ( *Hj* ).

### Molecular analyses

Genomic DNA was extracted from insect tho-races using the Insect Easy DNA Kit (EZNA) (Omega Bio-Tek, Norcross, GA) following the manufacturer’s protocol. The AFLP analysis followed the protocol developed by Vos et al. (1995) . Restriction‒ligation reactions were carried out in a total volume of 10 mL. A single reaction contained 500 ng of genomic DNA, 5 U *EcoR* I (MBI Fermentas, Vilnius, Lithuania) and 5 U *Tru* I ( *Mse* I isoschizomer) (Fermentas), 1.5 U T4 DNA Ligase (Fermentas), 1× T4 DNA Ligase Buffer (Fermentas), 0.05% BSA, 50 mM NaCl, 0.5 pmol/mL E-Adaptor, and 5 pmol/mL M-Adaptor. Reactions were carried out at room temperature overnight and then diluted five times with H _2_ O to obtain PCR matrices (pre-matrix DNA) for pre-selective amplification.

Pre-selective amplifications were carried out in 10 mL total volume. A pre-selective PCR mixture contained 2 mL pre-matrix DNA, 1× Qiagen Master Mix (Qiagen Taq PCR Master Mix Kit; Qiagen, Hilden, Germany), 0.5 mM E-primer (E+A), and 0.5 mM M-primer (M+C). Amplification was carried out using the following program: 72°C for 2 min, 20 cycles of 94°C for 20 sec, 56°C for 30 sec, and 72°C for 2 min, and finally 60°C for 30 min. A product of pre-selective PCR was diluted 20 times to obtain a PCR matrix for selective amplification (sel-matrix DNA).

Selective amplifications were carried out in 10 mL total volumes, consisting of 3 mL sel-matrix DNA, 1× Qiagen Master Mix, 0.5 mM FAM-labelled E-primer (E+ACA, E+ACC, E+ACG or E+ACT) and 0.5 mM M-primer (M+CAC, M+CAG or M+CAT) ( Table 2 ). PCR reaction was performed with the following program: 94°C for 2 min, 10 cycles of 94°C for 20 sec, 66°C ( **—** 1°C per cycle) for 30 sec, and 72°C for 2 min, 20 cycles of 94°C for 30 sec, 56°C for 30 sec, and 60°C for 30 min. Both pre-selective and selective amplifications were carried out using PTC200 thermocycler (BioRad, Hercules, CA).

**Table 2.**
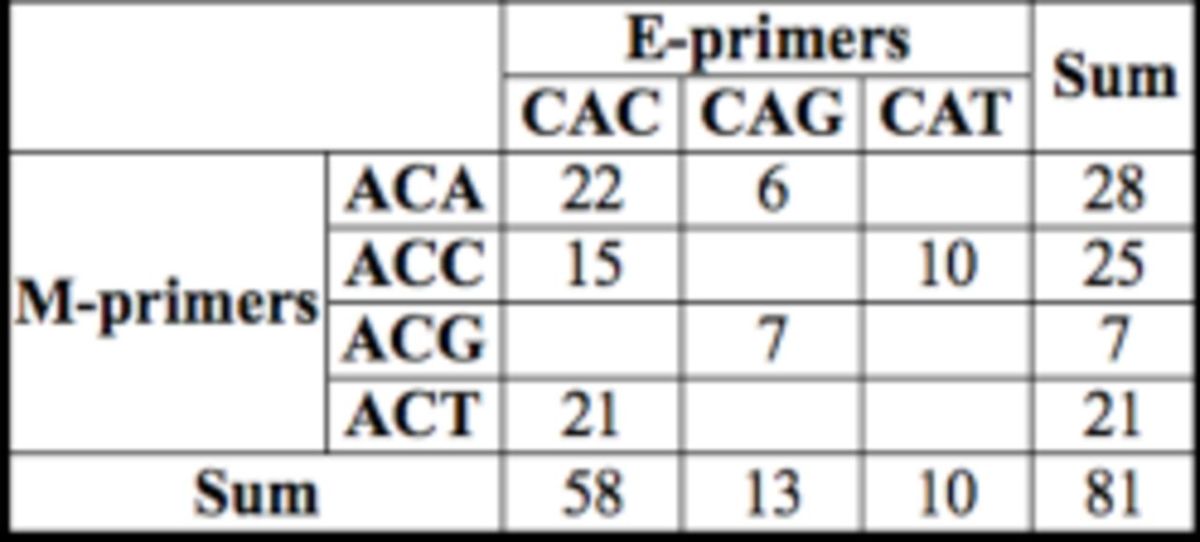
Number of suitable AFLP loci obtained from six combinations of primers.

The products of selective amplifications were sized using automated capillary sequencer ABI3130XL (Appllied Biosystems, Foster City, CA) and the manufacturer’s Genescan 3.7 software. AFLP profiles were then subjected to visual assessment to eliminate outlier samples in Genotyper 3.7 software (Applied Biosystems). Then, all peaks in a profile within the range 60-400 bp were automatically labelled, and bins were created based on all labelled peaks. Automatically created bins were visually checked to ensure that the bin was centered on the distribution of peaks within the bin. Bins with low polymorphism (3% < frequency of dominant haplotype <97%) were omitted from the further study. To reduce the occurrence of homoplasy ( Vekemans et al. 2002 ), bins with fragment-length distributions that overlapped with adjacent bins also were removed. Raw peak intensity data output from Genotyper were subsequently transformed into a binary data matrix with AFLPScore R-script (Whitlock et al. 2008).

### Statistical methods

#### Genetic variation.

For the purposes of analysis, it was assumed that the beetles collected from one sporocarp formed a distinct group of individuals (“population”). For each population, after estimating allelic frequencies with a Bayesian method, assuming a non-uniform prior distribution of allele frequencies (AFLP-SURV ver.1.0, Vekemans et al. 2002 ), the following statistics were computed: number and proportion of polymorphic loci at the 5% level, expected heterozygosity or Nei's gene diversity *(Hj* ), *and* Wright’s

#### Spatial genetic structure.

To test for the relationship between genetic and geographic pairwise distances, Mantel’s test was used to calculate the correlation between matrices of genetic distances [linearized and log-transformed geographic distances ( Slatkin 1995 ). This population-based approach, however, assumes an island model of population structure, which may not represent the true structure. Assigning sampled individuals to discrete groups even if the population is continuously distributed may result in failure to detect the real spatial genetic structure ( Schwartz and McKelvey 2008 ). Therefore, spatial autocorrelation analysis using a multilocus kinship coefficient was applied ( Hardy and Vekemans 1999 ). In contrast to population genetic estimators, which require averaging across populations, spatial autocorrelation makes no assumptions about the spatial scale of structuring in populations. The analysis was carried out for AFLP markers using SPAGeDi ver. 1.3 software ( Hardy and Vekemans 2002 ). To visualize the strength of spatial genetic structure, average pairwise kinship coefficients were plotted against distance classes. Because the relationship between kinship coefficients and distance typically takes log-linear form ( Rousset 2000 ), major changes in the spatial genetic structure are supposed to occur at closer distances. Therefore distance intervals for kinship comparisons were constructed in such a way that each successive interval was twice as large as the previous one. These classes were selected empirically to get detailed overview of the spatial genetic structure, as much as possible, while simultaneously obtaining the smoothed curves to get a sufficient number of pairwise comparisons. Average pairwise kinship coefficient per geographic distance interval was computed for the following distance classes: **∽** 0 m (within subpopulation, i.e., within one fungus sporocarp), 10 km, 20 km, 40 km, 80 km, 160 km, and >160 km. Within these distance classes, the numbers of pairwise comparisons among individuals were as follows: 624, 480, 861, 1161, 2759, 3079, and 216, respectively.

Confidence intervals around the average estimates of kinship coefficients for a given distance class were obtained from standard errors calculated by jack-knifing data over loci.

Under IBD, given drift-dispersal equilibrium, kinship is a linear function of logarithm of distance between individuals ( Rousset 2000 ). Therefore, to illustrate intensity of the spatial genetic structure, *Sp = - b1 /* (1 - *f*^(1^*))* index was estimated (Vekemans and Hardy 2004), where *b1* is a slope of a log-linear regression between observed kinship and a distance between individuals, and *f* is the average kinship estimated for the first distance class.

Spatial genetic structure (SGS) may arise because of IBD or spatial variation of selective forces affecting distribution of alleles of specific loci ( Beaumont and Balding 2004 ); therefore, only neutral loci were included in the SGS analyses. For this purpose, Mcheza software was used ( Antao and Beaumont 2011 ) to identify outlier loci, i.e., the loci considered to be candidates for directional and stabilizing selection. Mcheza implements popular DFDiSt method for dominant markers ( Beaumont and Nichols 1996 ) that relies on a Bayesian estimate of locus allele frequency ( Zhivotovsky 1999 ). Locus-specific values were estimated from a simulated distribution of 50,000 iterations using an infinite alleles model. The resulting distribution was used to find outliers. Outlier loci falling above the 0.99 quantile were considered putatively under directional selection, while under stabilizing selection when falling under 0.01. These markers were omitted from the further SGS and inbreeding analyses.

#### Inbreeding.

AFLP markers were analysed under the assumption of complete dominance (binary data). Hence, inference about inbreeding needs simultaneous estimation of allele frequencies and the inbreeding coefficient, based on phenotypes. The estimation was carried out using a Bayesian method introduced by Chybicki et al. (2011) and implemented in I4A computer program (available at request).

The method assumes that a sample of individuals, genotyped at AFLP loci, is randomly taken from a population characterized by an unknown average inbreeding coefficient *(F).* Each sampled individual is characterized by an individual inbreeding coefficient *(Fi)* that by assumption follows a beta distribution. Generally, a probability of observing a multilocus phenotype is a function of (unknown) allele frequencies and (unknown) individual inbreeding coefficient (as a proportion of identical by descent alleles in genotype of an individual). Using a Markov Chain Monte Carlo approach (mixed Gibbs-Metropolis algorithm), I4A estimates simultaneously marginal posterior distributions of the average inbreeding coefficient and allele frequencies given provided phenotypic data. Our estimates were obtained after 100,000 steps, after 10,000 burn-in steps. Because the method requires initial guesses on the priors, which are shape parameters of beta distribution *(a* and *b)* such, that *F = a / (a + b),* to avoid a dependence of final results on these guesses, analyses were conducted starting from three initial sets of parameters *(a* = *b* = {0.1,1,5}). Each set determines the same mean *(F* = 0.5) but implies very different shape (and variance) of the prior distribution.

## Results

### Genetic variation

From six combinations of primers in selective replication, 83 AFLP loci were reliably scored ( Table 1 ). Additional loci were present but were not scored, because of their ambiguity and inconsistency. After checking for neutrality with *F _ST_* –outlier method, only one marker was recognized as being under positive selection and one under stabilizing selection. These two loci were removed from further analyses, leaving 81 putatively neutral loci. On average, 86.2% loci were polymorphic within populations. The unbiased expected heterozygosity for all populations ranged from 0.302 ± 0.015 to 0.386 ± 0.023, with an average value of 0.347 ± 0.019 ( Table 2 ).

### Spatial genetic structure

**Figure 3.**
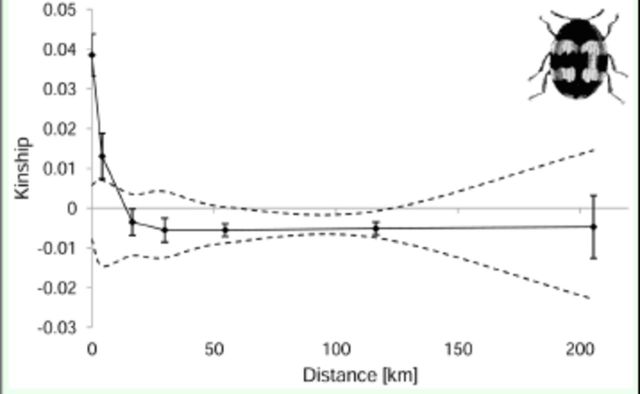
Average kinship coefficients between pairs of *Diaperis boleti* individuals plotted against the geographical distance. The observed value of pairwise kinship coefficient for mean value of each distance class and its 95% confidence interval obtained under the null hypothesis that genotypes are randomly distributed (dashed line) and standard error obtained by jack-knifing over loci (error bar) are shown. High quality figures are available online.

*FST* estimates between pairs of populations ranged between 0 and 0.111 with an average of 0.025 (SD 0.022). The Mantel test did not detect any significant correlation between matrices of genetic and geographic distances of populations *(R* = 0.09, *P* = 0.36). Nonetheless, more sensitive individual-oriented spatial autocorrelation analysis revealed that there was a relationship between genetic similarity of individuals and geographical distance. As expected based on the theory of isolation by distance, beetles that lived in close spatial proximity were more genetically similar than expected under a random distribution of genotypes. This pattern suggests a fine-scale genetic structure within *D. boleti* where proximate sites are genetically more alike than more distant ones. Average pairwise kinship coefficient was estimated to be 0.0386 (SD 0.005) for individuals collected from a single fruiting body of *L. sulphureus* . Kinship coefficient significantly exceeded zero for the two first distance classes ( Fig. 3 ). The point at which the autocorrelation curve first intercepts the x-axis (about 10 km) is expected to indicate an estimate of ‘patch’ size ( Smouse and Peakall 1999 ), i.e., overall extent of non-random genetic structure. In subsequent distance classes, the curve levelled off and values of kinship were indistinguishable from zero, except for the distance between 50 and 115 km, where values were significantly smaller than zero. The slope of the log-linear regression between distance and kinship was ‒0.0015 (SD 0.0007). The index *Sp* of the intensity of the spatial genetic structure was estimated as 0.0016 (SD 0.0007).

### Inbreeding

All three analyses executed with different prior distributions (see Methods) resulted in consistent estimates, which were slightly but significantly greater than zero (mean value 0.06; Table 3 ). Also, log-likelihood values for the three priors were not significantly different from one another, indicating that the estimates were stable regardless of the initial assumptions.

**Table 3.**
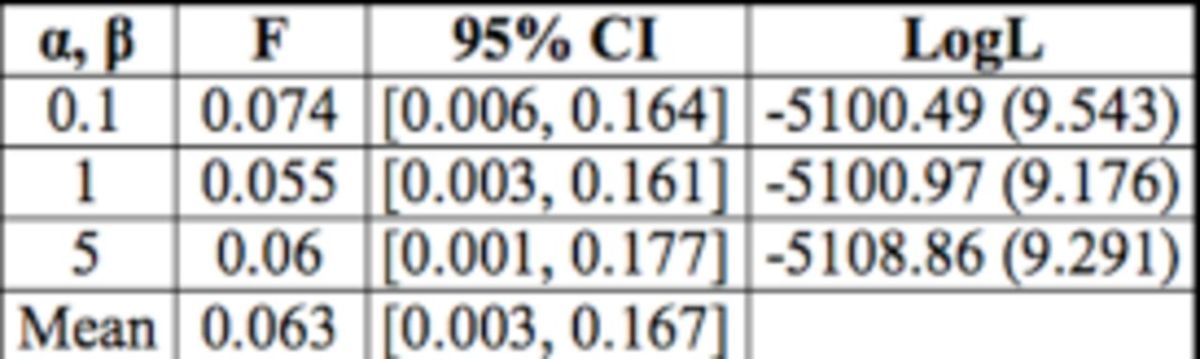
Average inbreeding coefficient ( *F* ) estimates for *Diaperis boleti* .

**α** , **β** - values of **α** and **β** parameters of the prior beta distribution used to infer about *F* ; 95 % CI - 95 % credible interval around *F* ; LogL - the average log-likelihood of data across the Markov Chain (standard deviation of LogL in parentheses)

## Discussion

Spatial genetic structure in the fungivorous beetle *D. boleti* is influenced by isolation by distance (IBD). Because intensive gene flow may be responsible for homogenizing spatial genetic structure, the presence of IBD could be surprising in a species with expected high mobility. It should be noted however that spatial clustering of related genotypes is also found in other mobile insects depending on ephemeral resources. A population survey of the forensically important black blow fly, *Phormia regina* (Meigen) (Diptera: Calliphoridae), found that adults arriving together at a carrion bait shared a much higher proportion of alleles than would be expected from a random sample ( Picard and Wells 2009 ). Spatial autocorrelation of allele frequency was found also in an Australian mosquito, *Ochlerotatus notoscriptus* Skuse (Diptera: Culicidae), indicating restricted gene flow and IBD effect ( Foley et al. 2004 ). These results show that even insects widely perceived as abundant have limited population sizes at local scale and occasional mating of related individuals is likely, as reflected by increased kinship coefficient and small but still significant average individual inbreeding.

Nonetheless, IBD in *D. boleti* —a mobile saproxylic beetle dependent on temporarily available supplies—is still weaker than in other previously studied saproxylic beetles dependent on more stable resources. Oleksa et al. (2012a) compared the spatial genetic structure of two ecologically and taxonomically related beetle species, *Osmoderma barnabita* Motschulsky (Coleoptera: Cetoniidae) and *Protaetia marmorata* (F.). Because the study was conducted within the same region as the present study, such comparisons are justified. Both *O. barnabita* and *P. marmorata* are exclusively associated with wood mould in tree hollows. Because large hollows develop in tree trunks during several decades, and some hollow trees could last for hundreds of years, durability of such a habitat is estimated for tens to hundreds of years ( Ranius et al. 2009 ), which translates into many generations of organisms living here. Many generations without intensive migration should in turn contribute to a strong genetic divergence between local populations and increased inbreeding. Furthermore, the differences between the populations of less abundant species should grow faster, as smaller populations are more prone to genetic drift. Comparison of the current study and the results of Oleksa et al. (2012a) are consistent with these predictions. Particularly strong IBD was observed in *O. barnabita (Sp* = 0.016 ± 0.002), while IBD in less specialised and more common *P. marmorata* was intermediate *(Sp* = 0.006 ± 0.003) between *O. barnabita and D. boleti (Sp =* 0.0016 ± 0.0007). In the case of *O. barnabita* and *P. marmorata,* observed differences in SGS were influenced not only by habitat stability but also species population characteristics, among which the most important seems to be niche breadth ( Oleksa et al. 2012a ). Similar conclusions apply to the overall pattern of genetic variation: the lowest heterozygosity was observed in *O. barnabita,* moderate in *P. marmorata,* and the highest in *D. boleti* (0.209, 0.290, and 0.347, respectively). It could be hypothesized that the weakening of gene flow may enhance the role of genetic drift at a local scale, therefore decreasing local effective population sizes and increasing the loss of genetic variation.

However, direct comparisons between species may be hindered by differences in responses to the habitat fragmentation, which is often regarded as the strongest factor affecting SGS. The spatial scale at which species perceive fragmentation varies with intrinsic species properties, like trophic level, dispersal ability, body size, and rarity ( Ewers and Didham 2006 ). For a given species, the influence of habitat fragmentation on SGS could be studied by comparing populations that occupy landscapes of different levels of fragmentation. For example, Knutsen et al. (2000) found that differentiation in the fungivorous beetle *Bolitophagus reticulatus* (L.) (Coleoptera: Tenebrionidae) in the fragmented forest was three times greater than in the continuous one, strongly indicating a genetic isolation effect of habitat fragmentation. However, it was unresolved whether landscape fragmentation increased genetic differentiation, because agricultural fields presented barriers to movement or more indirectly through gradually decreasing gene flow with increasing distance (i.e., IBD effect). Methods applied to infer IBD in *B. reticulatus* (correlation of matrices of pairwise population estimates of *FST* and geographic distances, i.e., Mantel test) did not find any statistically significant trend in genetic differentiation with distance and, hence, there was no clear evidence for IBD either in the fragmented or in the continuous area ( Knutsen et al. 2000 ). One may speculate that the applied methods (estimates at population level) were not sensitive enough to detect fine-scale pattern. In the present study, spatial autocorrelation analysis of kinship appeared to be a more sensitive approach than the Mantel test, probably because it makes full benefit of data from all pairs of individual locations across the sampled space ( Peakall et al. 2003 ). Therefore, the analyses of spatial autocorrelation analysis of kinship may be a particularly valuable approach in the case of organisms of conservation concern, and in consequence difficult to collect, like many saproxylic beetles. In the present study, the use of spatial autocorrelation found evidence of a weak spatial genetic structure at a finer scale, which remained undetected with the aid of the Mantel test.

## Conclusions

This is the first study of the spatial genetic structure of *D. boleti* using molecular markers. Compared with beetles living in more stable tree habitats, higher genetic diversity and weaker IBD pattern in a fungivorous species was demonstrated, in concordance with theoretical predictions. It was also demonstrated that the spatial autocorrelation analysis of kinship might be more sensitive in detecting fine-scale spatial genetic structure than the correlating matrices of genetic and geographic distances based on the Mantel test.

## Abbreviations

AFLP: amplified fragment length polymorphism
IBD: isolation by distance
SGS: spatial genetic structure
